# Amalgam x Composite Resin: supplies and restorative procedures more performed among Oral Health Teams in Brazil

**DOI:** 10.1590/0103-6440202305003

**Published:** 2023-01-15

**Authors:** Manoelito Ferreira Silva-Junior, Elis Carolina Pacheco, Natália Almeida Bastos-Bitencourt, Pollyana Kassia de Oliveira Borges, Marcia Helena Baldani

**Affiliations:** 1 Department of Health I, State University of Southwest Bahia (UESB), Jequie-BA, Brasil.; 2 Department of Dentistryof Universidade Estadual de Ponta Grossa (UEPG), Ponta Grossa-PR, Brasi.; 3 Department of Dentistry, Federal University of Juiz de Fora (UFJF-GV), Governador Valadares-MG, Brasil.; 4 Department of Public Health of Universidade Estadual de Ponta Grossa (UEPG), Ponta Grossa-PR, Brasil.

**Keywords:** Composite Resins, Dental Amalgam, Primary Health Care, Unified Health System

## Abstract

This study aimed to compare the availability of supplies and amalgam and composite resin restorations among Oral Health Teams (OHT) in Brazilian regions. Secondary data were extracted from Modules I and II of the 1^st^ (2012) and V and VI of the 2^nd^ (2014) and 3^rd^ cycle (2017) of the external evaluation of the National Program for Access and Quality in Primary Care^2^. The proportions between regions and cycles were compared using the Chi-square test with the z-test adjusted by the Bonferroni method (p<0.05). Among 2012, 2014, and 2017 there was a significant reduction in the proportion of OHT that performed amalgam restorations (87.5%, 89.2%, and 80.2%; p<0.001) and an increase in resin composite (92.5 %, 97.7%, and 99.0%; p<0.001), with the same trend in Brazilian regions (p<0.001). Amalgamator availability decreased among 2012 (99.0%), 2014 (98.4%) and 2017 (85.6%) (p<0.001). Amalgam availability was lower in 2017 (80.1%), compared to 2012 (87.5%) and 2014 (97.5%) (p<0.001). The availability of light curing decreased between 2012 (99.0%), 2014 (98.4%) and 2017 (85.6%) (p<0.001), being less available in the North (95.7%) (p<0.001). The light-curing resin increased between 2012 (94.1%), 2014 (96.6%) and 2017 (97.0%) (p<0.001), with no increase only in the North (p=0.134). While there was a reduction in supplies and amalgam restoration, there was an increase in supplies and resin composite restorations in the period evaluated in all Brazilian regions. However, regional disparities are still evident, with fewer supplies of restorative services in the North region.

## Resumo

Este estudo objetivou comparar a disponibilidade de fornecimentos e restaurações de amálgama e resina composta entre as Equipas de Saúde Oral (OHT) por regiões brasileiras. Os dados secundários foram extraídos dos módulos I e II do 1º (2012) e V e VI do 2º (2014) e 3º ciclo (2017) da avaliação externa do Programa Nacional de Acesso e Qualidade em Cuidados Primários. As proporções entre regiões e ciclos foram comparadas utilizando o teste Qui-quadrado com o teste z ajustado pelo método de Bonferroni (p<0,05). Entre 2012, 2014, e 2017 houve uma redução significativa na proporção de OHT que realizou restaurações de amálgama (87,5%, 89,2%, e 80,2%; p<0,001) e um aumento na resina composta (92,5%, 97,7%, e 99,0%; p<0,001), com a mesma tendência nas regiões brasileiras (p<0,001). A disponibilidade de amalgamadores diminuiu entre 2012 (99,0%), 2014 (98,4%) e 2017 (85,6%) (p<0,001). A disponibilidade de amálgama foi menor em 2017 (80,1%), em comparação com 2012 (87,5%) e 2014 (97,5%) (p<0,001). A disponibilidade de polimerização por luz diminuiu entre 2012 (99,0%), 2014 (98,4%) e 2017 (85,6%) (p<0,001), estando menos disponível no Norte (95,7%) (p<0,001). A resina fotopolimerizadora aumentou entre 2012 (94,1%), 2014 (96,6%) e 2017 (97,0%) (p<0,001), sem aumento apenas no Norte (p=0,134). Embora tenha havido uma redução nos fornecimentos e restauração de amálgamas, houve um aumento nos fornecimentos e restaurações de resina composta no período avaliado em todas as regiões brasileiras. Contudo, as disparidades regionais são ainda evidentes, com menos oferta de serviços de restauração na região Norte.

## Introduction

The Global Burden of Disease has demonstrated that the challenge of the population is represented by oral health. Although all preventive studies and improvements in dental science, untreated caries is still a global health problem. Developed countries have reduced inequalities in oral health with less burden of untreated caries while there is an increase of inequality in underdeveloped countries ^1^.

Modern management of dental caries consists in the diagnosis followed by intervention strategies focused on preventing and aiming to reverse the caries process. However, in extensive caries lesions, the treatment involves removing the demineralized tissue and its replacement with a filling restorative material.^2^ The development of adhesive techniques enabled a restorative approach to a more tooth-preserving one, without the need for mechanical retention ^2^ ^)^ and removal of the tissue affected by caries. However, evidence suggests that composite resins present a higher risk of secondary decay and higher failure rates than amalgam restorations ^3^.

On a large scale, as in public service, there is a logic of cost-effectiveness and scalability. Several factors must be considered, such as the cost of the material, the arsenal of supplies necessary, professional training to choose the ideal material in different approaches, clinical time for each treatment, aesthetic requirements involved. and the longevity of the material ^4^. In this sense, dental amalgam restorations have a high success rate and are the most cost-effective material in posterior tooth restorations. However, due to the anesthetics and the release of mercury from its composition, this material has declined use in Dentistry ^5^.

Brazil is the only country with more than 200 million inhabitants that has a universal health system. Since 1988, with the creation of the Unified Health System (Sistema Unico de Saúde - SUS, in Portuguese), several policies and programs have been created for its improvement. To attend integrality, in 2000, the Oral Health Teams (OHT) were included in the Family Health Strategy, offering dental services at Primary Health Care (PHC). In 2004, The National Oral Health Policy (Política Nacional de Saúde Bucal in Portuguese) (PNSB), in a process of expansion of primary oral health care and inclusion of Dentistry in secondary and tertiary care ^(^ ^6^.

Although SUS is a national policy, its financing occurs in a tripartite manner, that is, it depends on states and municipalities. Thus, the implementation and quality of oral health services vary according to loco-regional development. Brazil, a continental country, presents historical-socio-cultural differences that interfere in the epidemiology of oral diseases, as well as in the distribution and management of dental services. In PHC, the municipality becomes the main executor, and therefore, has greater decentralization and autonomy, which leads to greater variability in the form of implantation and in the quality of the dental service provided ^7^.

After the period of quantitative increase in the number of OHTs, there is an interest in achieving a qualitative improvement. The National Program for Improving Primary Care Access and Quality in Primary Health Care (Programa da Melhoria do Acesso e da Qualidade na Atenção Básica - PMAQ-AB - in Portuguese) was created for external assessments by independent academic institutions, obtaining a large repository of publicly available data that can be explored for research purposes ^8^.

Understanding that PHC resolution must be able to respond to 80-85% of the population's health problems ^6^, and the high prevalence of untreated caries in the Brazilian population ^(^ ^1^, cavity restorations are undoubtedly the most performed tanning procedure in this service. Several biological and technical factors can interfere with the choice of material used by dental surgeons ^9^, but other factors as the existence of supplies may partly justify the choice of the type of restorative material in public health services. Thus, this study aimed to compare the availability of supplies and amalgam and composite resin restorations among Oral Health Teams (OHT)in Brazilian regions.

## Materials and methods

### Study design

Time series was performed with secondary data from the External Evaluation of the Program for Improving Access and Quality of Primary Care (Programa de Melhoria dAcesso e da Qualidade da Atenção Básica - PMAQ-AB). The data were in the public domain and available by the Ministry of Health. The PMAQ-AB was created in 2011, aiming to encourage teams and managers to improve the quality of SUS services offered to the population and propose a set of strategies for better qualification, evaluation, and monitoring of the work developed by the health teams. So far, the PMAQ-AB has presented three cycles, each lasting two years, where the 1st cycle was performed in 2011-2012, 2nd cycle in 2013-2014, and 3rd cycle in 2015-2018.

All PMAQ-AB cycles were tripartite and coordinated by the Department of Primary Care (DPC) of the Ministry of Health, the National Council of Health Secretaries, and the National Council of Municipal Health Secretaries. For the external evaluation stage, there was the collaboration of Higher Education Institutions.

### Sample universe

The sample universe was represented by the OHT of the PHC that adhered to and received an external assessment in the 1^st^, 2^nd^ or 3^rd^ cycle of the PMAQ-AB. Adherence to the PMAQ-AB is voluntary and performed by the municipal management individually with the OHT that desires to participate. All OHTs were eligible, regardless of the respondent's professional category (dentist or oral health technician/assistant).

### Collection date

The external assessment of the PMAQ-AB was performed in cycles and in a multicentric way, under the responsibility of Higher Education Institutions from different Brazilian states. Also, the external assessment was divided into regions’ responsibility, which coordinated teams of previously selected independent interviewers. The evaluators were previously trained to collect data from professionals and users in the PHC facilities. Data were collected through electronic forms, using tablets, in addition to analyzing supporting documents, when necessary. The Informed Consent was presented to the participating professionals, and they were assured of the right to refuse to participate.

To facilitate the data collection process, the forms are divided into modules, which are divided according to the content and the respondent. In the first cycle, there were 3 modules: Module I (Structure of the Basic Health Unit), Module II (Health Teamwork process) and Module III (User). In the second and third cycles, in addition to the three modules included in the first, specific modules were included: Module IV (Family Health Support Center), Module V (Oral Health Structure), and Module VI (Teamwork process of Oral Health).

The present study used only modules referring to data collected with health professionals, and the selected variables were the dental supplies available to the OHT of Module V (2nd and 3rd Cycle) and referring to the work process of Module II (1st Cycle) and Module VI (2nd and 3rd Cycle).

### Variables

The dependent variables evaluated were related to dental supplies (instrumentals, permanent material, and bulk material) for amalgam and resin composite restorations, and the supply of both restorations (Suppl. 1).

The independent variables of the study were related to Brazilian geographic regions (North, Northeast, Midwest, Southeast, and South) and PMAQ-AB cycles (1^st^ Cycle (2012), 2^nd^ Cycle (2014), and 3^rd^ Cycle (2018)).

### Data analysis

The data obtained were analyzed using the Statistical Package for the Social Sciences (SPSS) 20.0 and presented as absolute (n) and relative (%) frequencies.

To compare the proportions between the variables studied (dependent) and the Brazilian geographic regions (independent variable) the Chi-square test (*α* = 0.05) was used, and to compare the cycles (years) (independent variable) was used the paired Cochran Q test (*α* = 0.05), both using the z test with Bonferroni adjustment (*α* = 0.05). Among the variables where the response options were 'Yes' or 'No', although the PMAQ-AB database tables only present response data referring to the 'Yes' category, the 'No' category was also considered in the analysis.

Equiplots-type graphics (https://www.equidade.org/equiplot_creator) were performed to visually present inequalities between Brazilian geographic regions.

## Results

**Figure 1:**
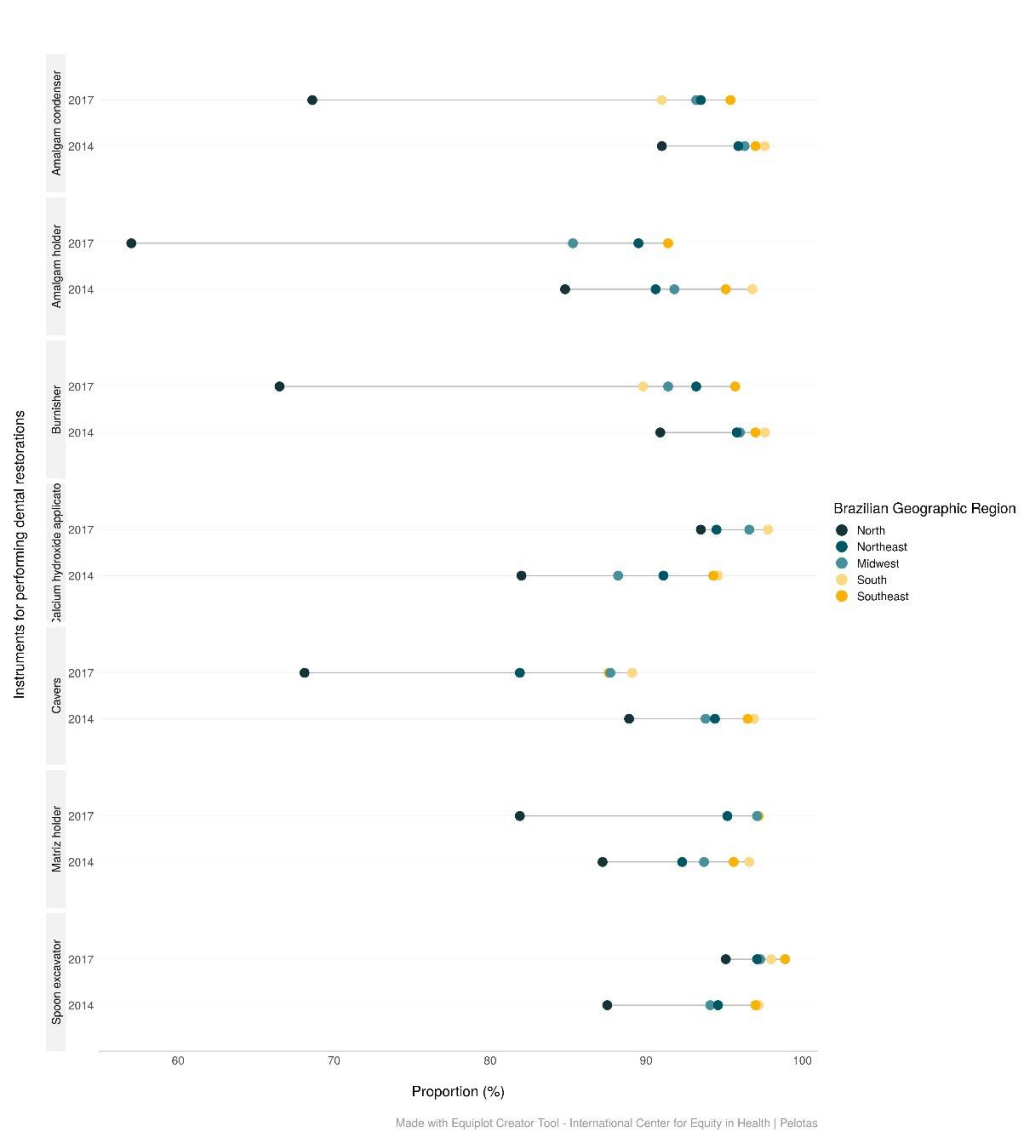
Equiplot of the availability of restorative instruments by Brazilian region and year. Brazil, 2014-2018.

In Brazil, the OHT showed high availability of permanent materials, instruments, and bulk materials, with a proportion above 90% for most of the supplies evaluated in the two cycles in which these items were included. The availability of matrix holders, dentin excavators, and resin composite spatula increased in Brazil (p<0.001) and in Brazilian regions (p<0.001), except the North region which reduced the availability of matrix holders (p <0.001). In the country, there was a reduction in all the other instruments analyzed, and more accentuated in the proportion of the amalgam holder, condensers, burnishers, and carvers (p<0.001). The South and Southeast regions presented the highest number of instruments available, and therefore, the increase was less expressive or unchanged in the evaluated time. The North showed a lower proportion compared to the other regions in each cycle, in addition to a further reduction between the years 2014 and 2018 (Table 1) (Figure 1).

**Table 1:** Availability distribution of instruments for performing dental restorations by Oral Health Teams, according to Brazilian geographic regions and years. Brazil, 2012-2018.

		Brazilian Geographic Region					
Variables	Brazil	South	Southeast	Midwest	Northeast	North	p-value*
	n (%)	n (%)	n (%)	n (%)	n (%)	n (%)	
Amalgam holder							
2014 (n=15052)	13918 (92.5)A	2006 (96.8)Aa	3710 (95.1)Ab	1194 (91.8)Ac	6381 (90.6)Ac	627 (84.8)Ad	<0.001
2018 (n=20301)	17592 (86.7)B	2267 (85.3)Ba	4637 (91.4)Bb	1440 (85.3)Ba	8391 (89.5)Bc	857 (57.0)Bd	<0.001
p-value**	<0.001	<0.001	<0.001	<0.001	0.015	<0.001	
Matriz holder							
2014 (n=15739)	14724 (93.6)A	2174 (96.6)Aa	3800 (95.6)Aa	1292 (93.7)Ab	6625 (92.3)Ab	833 (87.2)Ac	<0.001
2018 (n=20301)	19308 (95.1)B	2580 (97.1)Aa	4932 (97.2)Ba	1640 (97.1)Ba	8925 (95.2)Bb	1231 (81.9)Bc	<0.001
p-value**	<0.001	0.375	<0.001	<0.001	<0.001	<0.001	
Spoon excavator							
2014 (n=15714)	14938 (95.1)A	2188 (97.2)Aa	3855 (97.0)Aa	1281 (94.1)Ab	6678 (94.6)Ab	936 (84.5)Ac	<0.001
2018 (n=20301)	19805 (97.6)B	2606 (98.0)Aa	5018 (98.9)Bb	1643 (97.3)Ba	9108 (97.1)Ba	1430 (95.1)Bc	<0.001
p-value**	<0.001	0.064	<0.001	<0.001	<0.001	<0.001	
Calcium hydroxide applicator							
2014 (n=15857)	15031 (94.8)	2181 (97.1)a	3802 (95.9)a,b	1291 (94.2)b,c	6826 (94.5)c	931 (88.4)d	<0.001
2018 (n=20301)	19160 (94.4)B	2576 (96.9)Aa	4799 (94.6)Bb	1598 (94.6)Bb	8830 (94.2)Bb	1357 (90.3)Bc	<0.001
p-value**	<0.001	0.578	<0.001	0.02	<0.001	0.02	
Amalgam condenser							
2014 (n=15147)	14574 (96.2)A	2074 (97.6)Aa	3827 (97.0)Aa,b	1286(96.3)Aa,b	6687 (95.9)Ab	700 (91.0)Ac	<0.001
2018 (n=20301)	18631 (91.8)B	2419 (91.0)Ba	4839 (95.4)Bb	1574 (93.2)Ba,c	8768 (93.5)Bc	1031 (68.6)Bd	<0.001
p-value**	<0.001	<0.001	<0.001	<0.001	<0.001	<0.001	
Burnisher							
2014 (n=15547)	14945 (96.1)A	2109 (97.6)Aa	3878 (97.0)Aa	1303(96.0)Aa,b	6928 (95.8)Ab	727 (90.9)Ac	<0.001
2018 (n=20301)	18518 (91.2)B	2386 (89.8)Ba	4854 (95.7)Bb	1543 (91.4)Ba,c	8735 (93.2)Bc	1000 (66.5)Bd	<0.001
p-value**	<0.001	<0.001	0.001	<0.001	<0.001	<0.001	
Resin composite spatula							
2014 (n=15238)	13946 (91.5)A	2119 (94.6)Aa	3561 (94.3)Aa	1158 (88.2)Ab	6246 (91.1)Ac	862 (82.0)Ad	<0.001
2018 (n=20301)	19293 (95.0)B	2600 (97.8)Ba	4797 (94.5)Ab	1631 (96.6)Ba	8859 (94.5)Bb	1406 (93.5)Bb	<0.001
p-value**	<0.001	<0.001	0.564	<0.001	<0.001	<0.001	
Cavers							
2014 (n=14906)	14147 (94.9)A	2107 (96.9)Aa	3727 (96.5)Aa	1232 (93.8)Ab	6290 (94.4)Ab	791 (88.9)Ac	<0.001
2018 (n=20301)	16995 (83.7)B	2367 (89.1)Ba	4445 (87.6)Ba	1482 (87.7)Ba	7677 (81.9)Bb	1024 (68.1)Bc	<0.001
p-value**	<0.001	<0.001	<0.001	<0.001	<0.001	<0.001		

*Lowercase letters represent statistical difference between Brazilian geographic regions per line (p<0.05).

**Capital letters represent statistical difference between cycles (years) per column (p<0.05).

**Figure 2:**
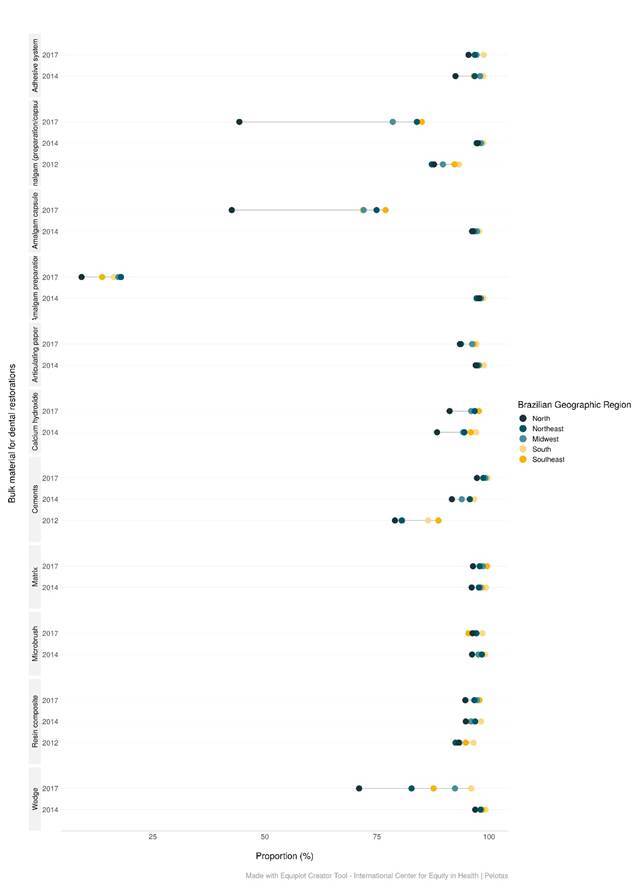
Equiplot of the availability of bulk materials used in restorative procedures by Oral Health Teams, according to Brazilian region and year. Brazil, 2012-2018.

**Figure 3:**
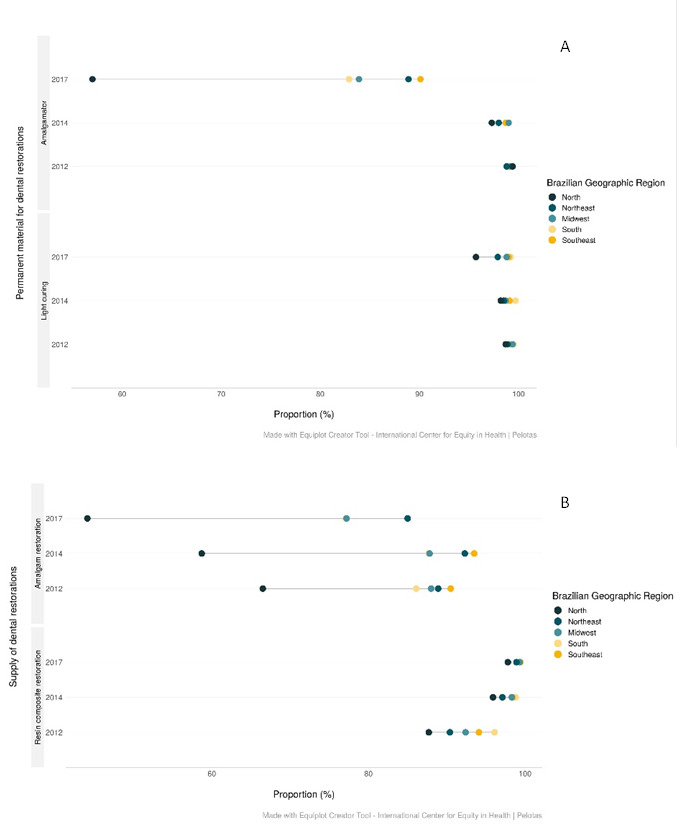
Equiplot of the availability of (a) permanent materials used in restorative procedures and (b) supply of restorations by Oral Health Teams, according to Brazilian region and year. Brazil, 2012-2018.

Among bulk materials, there was an increasing trend in Brazil between 2012 (90.3%) and 2014 (97.5%), and after a reduction in 2018 (80.1%) in amalgam for preparation or capsule (p<0.001). The reduction was more expressive in the amalgam for preparation between 2014 (96.8%) and 2018 (15.9%) (p<0.001). The reduction occurred in all Brazilian regions and was more expressive in the North region (Table 2) (Figure 2).

**Table 2:** Availability distribution of bulk material for dental restorations by Oral Health Teams, according to Brazilian geographic regions and years. Brazil, 2012-2018.

Variables	Brazil n (%)	Brazilian Geographic Region					p-value*
		South n (%)	Southeast n (%)	Midwest n (%)	Northeast n (%)	North n (%)	
Amalgam (preparation or capsule)							
2012 (n=19160)	17292(90.3)A	3242(93.3)Aa	5906(92.3)Aa	1424(89.7)Ab	6000(87.2)Ab	720(87.7)Ab	<0.001
2014 (n=13934)***	13588(97.5)B	1890(98.7)Ba	3548(97.3)Bb	1199(98.2)Ba,b	6319(97.2)Bb	632(97.5)Ba,b	0.002
2018 (n=20301)***	16256(80.1)C	2090(78.6)Ca	4311(85.0)Cb	132678.5)Ca	7863(83.9)Cb	666 44.3)Cc	<0.001
p-value**	<0.001	<0.001	<0.001	<0.001	<0.001	<0.001	
Amalgam capsule							
2014 (n=11065)	10796 (97.6)A	1605(98.8)Aa	2848(97.3)Ab	1021(98.2)Aa,b	4741(97.2)Ab	581(97.8)Aa.b	0.003
2018 (n=20301)	14689 (72.4)B	1905(71.7)Ba	3903(76.9)Bb	1216(72.0)Ba,c	7025(74.9)Bb,c	640(42.6)Bd	<0.001
p-value**	<0.001	<0.001	<0.001	<0.001	<0.001	<0.001	
Amalgam preparation							
2014 (n=4291)	4153(96.8)A	489(97.8)Aa	1106(96.7)Aa	320(97.3)Aa	2113(96.6)Aa	125(96.2)Aa	0.658
2018 (n=20301)	3237(15.9)B	434(16.3)Ba	693(13.7)Bb	293(17.3)Ba	1680(17.9)Ba	137(9.1)Bc	<0.001
p-value**	<0.001	<0.001	<0.001	<0.001	<0.001	<0.001	
Resin composite							
2012 (n=21917)	20621 (94.1)A	3986(96.5)Aa	6647(94.8)Ab	1688(93.4)Ab,c	7096(92.5)Ac	1224(93.2)Ab,c	<0.001
2014 (n=16114)	15569 (96.6)B	2239(98.2)Ba	3850(95.9)Bb	1338(96.0)Bb,c	7102(96.9)Bc	1041(94.8)Ab	<0.001
2018 (n=20301)	19700 (97.0)B	2603(97.9)Ba	4963(97.8)Ca	1643(97.3)Ba,b	9068(96.7)Bb	1423(94.7)Ac	<0.001
p-value**	<0.001	<0.001	<0.001	<0.001	<0.001	0.134	
Adhesive system							
2014 (n= 15974)	15441 (96.7)A	2248(98.7)Aa	3851(96.6)Ab	1331(96.0)Ab	7012(96.8)Ab	999(92.5)Ac	<0.001
2018 (n= 20301)	19711 (97.1)B	2626(98.8)Aa	4932(97.2)Ab	1642(97.2)Ab,c	9077(96.8)Ab,c	1434(95.4)Bc	<0.001
p-value**	0.019	0.825	0.121	0.054	0.952	0.002	
Cements							
2012 (n=21296)	17939 (84.2)A	3515(86.4)Aa	6162(88.7)Ab	1403(80.6)Ac	5899(80.5)Ac	960(79.0)Ac	<0.001
2014 (n=15187)	14499 (95.5)B	2134(96.7)Ba	3719(95.8)Ba,b	1224(93.9)Bb,c	6554(95.7)Ba	868(91.7)Bc	<0.001
2018 (n=19911)***	19689 (98.9)C	2633(99.7)Ca	4978(99.2)Cb	1631 (99.2)Ca,b	9027(98.7)Cb	1420(97.3)Cc	<0.001
p-value**	<0.001	<0.001	<0.001	<0.001	<0.001	<0.001	
Calcium hydroxide cement							
2018 (n=20301)	19160(94.4)	2576(96.9)a	4799(94.6)b	1598(94.6)b	8830(94.2)b	1357(90.3)c	<0.001
Glass ionomer cement							
2018 (n=20301)	18821(92.7)	2587(97.3)a	4789(94.4)b	1565(92.7)b,c	8566(91.4)c	1314(87.4)d	<0.001
Zinc oxide cement							
2018 (n=20301)	15886(78.3)	2086(78.5)a	4345(85.6)b	1310(77.6)a	7124(76.0)a	1021(67.9)c	<0.001

*Lowercase letters represent statistical difference between Brazilian geographic regions per line (p<0.05).

**Capital letters represent statistical difference between cycles (years) per column (p<0.05).

***Variables where adjustment was necessary for data comparability.

**Table 3:** Availability distribution of bulk material for dental restorations by Oral Health Teams, according to Brazilian geographic regions and years. Brazil, 2012-2018.

Variables	Brazil n (%)	Brazilian Geographic Region					p-value*
		South n (%)	Southeast n (%)	Midwest n (%)	Northeast n (%)	North n (%)	
Cementation cement							
2018 (n=20301)	15705 (77.4)	2011(75.7)a,b	4171(82.2)c	1251(74.1)b	7318(78.0)a	954(63.5)d	<0.001
Wedge							
2018 (n=20301)	17380 (85.6)B	2552(96.0)Ba	4446(87.6)Bb	1561(92.4)Bc	7754(82.7)Bd	1067(71.0)Be	<0.001
Matrix							
2014 (n=16009)	15697 (98.1)A	2269(99.3)Aa	3922(98.4)Ab	1370(98.0)Ab,c	7117(97.7)Ab,c	1019(96.1)Ac	<0.001
2018 (n=20301)***	19987 (98.5)B	2642(99.4)Aa	5048(99.5)Ba	1665(98.6)Ab	9183(97.9)Ac	1449(96.4)Ad	<0.001
p-value**	0.003	0.808	<0.001	0.211	0.418	0.717	
Polyester matrix							
2018 (n=20301)	19423(95.7)	2620(98.6)a	4905(96.7)b	1621(96.0)b,c	8869(94.6)c,d	1408(93.7)d	<0.001
Metallic matrix							
2018 (n= 20301)	19689(97.0)	2627(98.8)a	4968(97.9)b	1655(98.0)a,b	9028(96.3)c	1411(93.9)d	<0.001
Microbrush							
2014 (n=15377)	15105(98.2)A	2228(99.1)Aa	3577(98.2)Aa,b	1301(97.6)Ab,c	6970(98.4)Aa,b	1029(96.2)Ac	<0.001
2018 (n=20301)	19659(96.8)B	2619(98.5)Aa	4842(95.4)Bb	1641(97.2)Ac	9109(97.1)Bc	1448(96.3)Ab,c	<0.001
p-value**	<0.001	0.09	<0.001	0.453	<0.001	0.82	
Articulating paper							
2014 (n=15025)	14679(97.7)A	2207(98.8)Aa	3787(97.8)Ab	1337(97.7)Aa,b	6382(97.4)Ab	966(97.0)Ab	0.001
2018 (n=20301)	19302(95.1)B	2584(97.2)Ba	4901(96.6)Ba	1625(96.2)Ba	8787 (93.7)Bb	1405(93.5)Bb	<0.001
p-value**	<0.001	<0.001	0.001	0.016	<0.001	<0.001	
Polishing strips polyester							
2018 (n=20301)	19739(97.2)	2626(98.8)a	4962(97.8)b	1655 (98.0)a,b	9053(96.5)c	1443(96.0)c	<0.001
Metallic strip							
2018 (n=20301)	19383(95.5)	2590(97.4)a	4844(95.5)b	1643 (97.3)a	8914(95.1)b	1392(92.6)c	<0.001

*Lowercase letters represent statistical difference between Brazilian geographic regions per line (p<0.05).

**Capital letters represent statistical difference between cycles (years) per column (p<0.05).

***Variables where adjustment was necessary for data comparability.

The percentage of availability of light-curing resin increased in Brazil between 2012 (94.1%) and 2014 (96.6%), but without differing between 2014 and 2018 (97.0%), as happened in the South, Central and West and Northeast (p<0.001). While in the Southeast an increase in light-curing resin was observed between cycles (p<0.001), in the North it remained unchanged (p=0.134), and with smaller proportions compared to other Brazilian regions (p<0.001). Adhesive system availability increased in Brazil (p<0.001), only as a result of the significant increase in the North region (p=0.002), since all other regions remained with equal proportions (p>0.05) Cements presented an increase in the proportion of availability in all Brazilian regions (p<0.001). In 2018, calcium hydroxide cement was the most available (94.4%), and cementation cement was the least available (77.4%) in Brazil (Table 2) (Figure 2).

 Wedge, microbrush, and articulating paper were reduced between 2014 and 2018 (p<0.001). The matrix was the only bulk material that increased between 2014 and 2018 (p<0.001). In 2018, the metallic matrix (97.0%) was more available than the polyester matrix (95.7%). However, in the same year, polishing strips of polyester (97.2%) was more available than a metallic strip (95.5%) (Table 3) (Figure 2). Regional inequalities remained in the various items of bulk material, with better proportions in the South and Southeast regions, and lower in the North (p<0.001) (Table 2 and 3) (Figure 2).

Among the permanent materials, there was a successive reduction between OHT with the availability of an amalgamator and a curing light in Brazil (p<0.001). While in 2012, 99.0% of the OHT presented an amalgamator and 99.1% a light curing. In 2018, an amalgamator was available for 85.6% and the light curing for 98.2% of the OHT. Inequalities between regions remain for both materials in most years evaluated (p<0.05). The reduction in the proportion of amalgamator occurred for all Brazilian regions (p<0.001), but it was more pronounced in the North (57.0%) (2018). The availability of light curing showed an unchanged proportion over time in the South, Southeast, Midwest (p>0.05), and reduced in the Northeast and North (p<0.001) (Table 4) (Figure 3a).

**Table 4:** Availability distribution of permanent material for dental restorations by Oral Health Teams, according to Brazilian geographic regions and years. Brazil, 2012-2018.

Variables	Brazil n (%)	Brazilian Geographic Region					p-value*
		South n (%)	Southeast n (%)	Midwest n (%)	Northeast n (%)	North n (%)	
Amalgamator							
2012 (n=20383)	20184 (99.0)A	3767(99.4)Aa	6652(99.0)Aa,b	1664(9.,2)Aa,b	7236(98.8)Ab	865(99.4)Aa,b	0.029
2014 (n=14221)	13990 (98.4)B	1815(99.0)Aa	3682(98.7)Aa,b,c	1243(99.0)Aa,c	6612(98.0)Bb,c	638(97.3)Bb	0.001
2018 (n=22046)	18875 (85.6)C	2386(82.9)Ba	5052 (90.1)Bb	1499(83.9)Ba	8990(88.9)Cb	948(57.0)Cc	<0.001
p-value**	<0.001	<0.001	<0.001	<0.001	<0.001	<0.001	
Light curing							
2012 (n=21671)	21485 (99.1)A	4049(99.5)Aa	6861 (99.2)Aa,b	1769(99.4)Aa,b	7526 (98.9)Ab	1280(98.7)Ab	0.010
2014 (n=15529)	15339 (98.8)B	2008(99.7)Aa	3926 (99.1)Aa.b	1368 (98.7)Ab	7009 (98.5)Bb	1028(98.2)Ab	<0.001
2018 (n=22046)	21659 (98.2)C	2856(99.2)Aa	5549 (99.0)Aa	1765(98.8)Aa,b	9897 (97.9)Cb	1592(95.7)Bc	<0.001
p-value**	<0.001	0.088	0.470	0.112	<0.001	<0.001	

*Lowercase letters represent statistical difference between Brazilian geographic regions per line (p<0.05).

**Capital letters represent statistical difference between cycles (years) per column (p<0.05).

The supply of amalgam restorations decreased over the years, from 87.6% (2012) to 80.2% (2018) (p<0.001), and a reduction in all Brazilian regions (p<0.001). The North, with the lowest proportion since 2012 (66.5%), had the higher reduction in 2018 (44.1%). There was an increase in resin composite restorations (p<0.001), from 92.5% (2012) to 99.0% (2018) in all Brazilian regions (p<0.001). Inequalities in 2018 were less expressive between the region with the highest availability (Southeast - 97.8%) and the lowest proportion (North - 97.3%). Provisional restorations had a high proportion of supply in 2012 (90.8%), and glass ionomer cement restorations in 2018 (97.0%) (Table 5) (Figure 3b).

**Table 5:** Distribution of the supply of dental restorations performed by Oral Health Teams, according to Brazilian geographic regions and years. Brazil, 2012-2018.

Variables	Brazil n (%)	Brazilian Geographic Region					p-value*
		South n (%)	Southeast n (%)	Midwest n (%)	Northeast n (%)	North n (%)	
Amalgam restoration							
2012 (n=12562)	10987(87.6)A	1728(86.1)Aa	3657(90.5)Ab	816 (88.0)Aa,b	4249 (88.9)Ab	537 (66.5)Ac	<0.001
2014 (n=18333)	16349(89.2)B	2257(87.7)Aa	4728(93.5)Bb	1392 (87.7)Aa	7206 (92.3)Bb	766 (58.7)Bc	<0.001
2918 (n=22993)	18450(80.2)C	2313(77.1)Ba	5197(85.0)Cb	1493 (77.2)Ba	8681 (85.0)Cb	766 (44.1)Cc	<0.001
p-value**	<0.001	<0.001	<0.001	<0.001	<0.001	<0.001	
Resin composite restoration							
2012 (n=12562)	11616(92.5)A	1930(96.1)Aa	3800(94.1)Ab	857 (92.4)Ab,c	4320 (90.4)Ac,d	708 (87.7)Ad	<0.001
2014 (n=18333)	17920(97.7)B	2544(98.8)Ba	4979(98.4)Ba	1560(98.3)Ba,b	7586 (97.1)Bb,c	1251(95.9)Bc	<0.001
2018 (n=22993)	22767(99.0)C	2981(99.3)Ba.b	6074(99.4)Cb	1919 (99.3)Ca,b	10096 (98.9)Ca	1697(97.8)Cc	<0.001
p-value**	<0.001	<0.001	<0.001	<0.001	<0.001	<0.001	
Provisional restoration							
2012 (n=12562)	11402 (90.8)	1912 (95.2)a	3744 (92.7)b	826 (89.1)c	4253 (89.0)c	667 (82.5)d	<0.001
Restoration of Glass ionomer cement							
2018 (n=22993)	22310 (97.0)	2959 (98.6)a	6027 (98.6)a	1873 (96.9)b	9798 (96.0)b	1653 (95.2)b	<0.001

*Lowercase letters represent statistical difference between Brazilian geographic regions per line (p<0.05).

**Capital letters represent statistical difference between cycles (years) per column (p<0.05).

## Discussion

The present study showed a tendency to reduce the proportion of teams that perform amalgam restorations and a higher supply of resin composite restorations. This phenomenon has been described in the literature ^10^, although a systematic review and meta-analysis suggest that restorations in posterior teeth with resin composite had higher rates of failure when compared to amalgam. However, public surveys studies have shown that 40% of resin composite restorations in premolar teeth and 41% in molar teeth have survived without re-intervention at 15 years, considering that other factors may influence this survival rate such as patient age, dentist age, the patient's need for treatment and the type of restoration ^11^ ^,^ ^12^.

The amalgam replacement process is evident in Brazil and worldwide, and several factors can influence this process. The literature states that amalgam restorations release mercury over time, a highly toxic compound ^13^. In addition, the socio-environmental conditions related to the disposal of amalgam, and since the Minamata Convention, in 2013, there has been an effort to reduce the use of mercury in the world ^14^. The Brazilian Health Regulatory Agency, through Directors' Collegiate Resolution N^o^ 173 of September 15, 2017, as of January 1, 2019, it prohibited the manufacture, import, marketing and use, in health services, of mercury and powder for amalgam alloy in non-encapsulated form ^15^. Therefore, the cost of encapsulated amalgam may have become another limiting factor in the choice of this material in the public health service. In addition, in Brazil, the waste generator is responsible for its management, and therefore, is responsible for its final disposal. The absence of specialized companies capable of carrying out the reverse logistics of disposal has become an additional difficulty for health services to ensure occupational and environmental health related to mercury contamination.

In the present study, even with the high availability of supplies related to resin composite since the first cycle of the PMAQ-AB (2011-2012), there was an increase in the availability of instruments and bulk material, such as resin composite and spatulas, respectively. The reduction in the availability of light-curing was not very expressive and only significant in the North and Northeast regions. This reduction can be explained by the increase in the adherenceto OHT evaluated in the last cycle, with the participation of less qualified health units. In addition, the permanent material may be undergoing maintenance, considering that only the equipment in use condition at the time of collection was evaluated. On the other hand, regarding the availability of dental supplies related to amalgam restorations, there was a reduction over time both for specific instruments, such as burnishers, condensers, sculptors, amalgam holders, and also in bulk and permanent materials, as amalgam and amalgamator.

The lower amalgam supplies availability and more availability of supplies and instruments related to the resin composite favored the realization of restorations. In addition, it is necessary to consider that these restorations showed an increase in the OHT supply over time in all Brazilian regions. The PNSB after overcoming the barrier of institutionalization as a public health policy presented a joint effort to replace scrapped equipment and expand the physical infrastructure to improve installed capacity ^6^. This replacement process may have enhanced the concept of `new` technologies, and therefore emphasis on resin composite restorations.

Regional inequalities in the distribution of supplies remained; however, there is a reduction in the distance from these disparities, especially concerning the supply of resin composite restorations by OHT. The reduction of inequalities between Brazilian regions may be the result of a prioritization of the restorative procedures in solving a high demand in Primary Health Care ^6^, resulting from the impact of oral health conditions, and dental caries ^1^. Oral health surveys in Brazil between 1986 and 2010 showed that there was an improvementadults’ oral health. This age group was excluded in oral health policies before the PNSB, since 2004 ^16^. This fact can be explained by the increase in the restored tooth component in the composition of the decayed, missing, and filled teeth index in the last epidemiological survey of oral health in Brazil. It was observed improvements in sociodemographic conditions until the year of the last oral epidemiologic survey and the incorporation of fluoride in public water supply and toothpastes ^16^. In addition, it was the consolidation of the SUS and the PNSB with the increase in the number of OHT teams that are factors that lead to the prioritization of restorations over extractions.

In the present study, the results showed the persistence of regional health inequalities in Brazil, as shown in the literature ^17^. North region showed the lowest availability of dental supplies and equipment, including amalgam restorations. Considering the lower availability of supplies for adhesive restorations in the North region, which could compromise the performance of restorations, the reduction of supplies for amalgam restorations becomes contradictory. Although there are several controversies regarding the use of amalgam, studies prove its effectiveness, low cost, and longevity, considering it a safe option based on clinical and economic reasons ^18^. The North region is known not only for the worst sociodemographic indicators but additional difficulties such as large territorial extension and mobility difficulties, which reflect in health care, low dentistry supply, and dentists’ retention. Therefore, it is important to have public health policies that consider the different contexts, demands, and territorial and social configurations, which are fundamental to strengthening the SUS.

Composite resins have taken up more space in teaching environments, which certainly influences the professional choice regarding these restorative materials ^(^ ^19^, it is important to recognize that amalgam-free dental schools are a reality in several countries. Resin composite has advantages related to aesthetics, biocompatibility, and conservative preparation, however, it is sensitive to technical steps and therefore strongly influenced by the professional ^20^. Adhesive restorations have more steps that are clinical and depend on more material resources and therefore may influence in the reduced longevity ^21^. However, self-adhesive/universal adhesives and bulk-fill composites have simplified restorative procedures, although future studies should consider whether these materials are included in public service.

The greater use of Glass Ionomer Cement (GIC) for definitive restorative purposes is related to adhesion to the dental substrate, thermal capacity close to the dental substrates, fluoride release, and resistance to occlusal forces, and therefore presents a high percentage of survival in posterior teeth, which are considered the most affected teeth by caries ^(^ ^22^. In the present study, the high availability and the offer of GIC restoration were evaluated only in the last cycle (2018), which makes comparability difficult. However, there is a hypothesis that the GIC may be becoming the material that will replace amalgam, especially the Atraumatic Restoration Technique (ART) ^4^. When considering the low cost and effectiveness of ART, there is a need to maintain the availability of both supplies, so that the clinical decision of the restorative technique is not related to the unavailability or absence of supplies.

Another highlight is related to the increase in the aesthetic demand of patients, including the replacement of amalgam restorations with resin composite ^23^. The professional must differentiate the unsatisfactory restorations from which they must be replaced, in addition to restorations that can be repaired, avoiding invasive treatments and placing the patient in a repetitive restorative cycle ^20^. It should be considered that the repair of amalgam restorations, when properly indicated, is a minimally invasive practice with satisfactory longevity.

In addition to the technical point of view, the type of material used in dental restorations is associated with social and racial factors and becomes a marker of social inequality in Brazil ^24^. In public service, the wide use of amalgam has been justified by its excellent physical and mechanical properties, good performance, low cost, and longevity ^22^. However, in the private service, there is no consensus regarding the clinical applicability of amalgam, due to the improvement in the mechanical properties of composite resins and the aesthetics ^22^. A cohort study performed in the Southern region of Brazil showed that social classes marked by skin color in Brazil determine the type of service used (public or private), and thus the material used in restoration can confirm the social gap that still exists in clinical practices ^(^ ^23^. The white individuals with high levels of education have a higher probability to have their posterior amalgam restorations replaced by resin composite ^23^.

The limitations of the study are related to data collection with secondary sources. The PMAQ-AB is a program with voluntary adhesion by OHT, and therefore, the database can present results among teams with better performance, which may not reflect the Brazilian reality. This aspect may be inherent mainly in the first cycle, where there is an explicit difference in the number of responses for infrastructure (available materials) and work process (performance of procedures) items. This happened because together with the PMAQ-AB in the first cycle, there was a limitation of adherence not exceeding 50% of the number of OHTs in the municipality. Thus, there may have been an adhesion and evaluation about the performance of procedures of the best OHT, since the team of interest by the municipality was registered. This issue is better understood from the second cycle, with the end of the restriction the initial adherence, and substantial increase in the proportion of OHT participants, and adherence above 90% in the third cycle. Therefore, it can give higher reliability to the results presented when considering a possible quality in the non-participating OHTs, which are a minority.

In addition, the study presents a limitation in the comparability of the questions between the cycles, whether in terms of quantity, format, or possibility of answers. In order to minimize comparison errors, care was taken in the preparation of the database and in the detailed presentation of the adaptations in the comparability process. For the measurement of inequalities, the maintenance of coherence in the assessment instruments is essential when aiming for a universal, equitable, and integral system.

The PMAQ-AB is a valuable instrument for maintaining the quality of oral health in Primary Care. However, the program has currently been replaced by another evaluation proposed by the Federal Government, the Previne Brasil ^25^. The change means a substantial loss of quality indicators for the care provided, which were achieved over time and helped to consolidate the quality of the service provided by the Unified Health System. This fact makes it impossible to effectively compare the cycles in the future. In addition to the loss in the evaluation of the quality of care, the lack of comparison between cycles impairs the effective and equal distribution of supplies in the PHC facilities. Therefore, without these indicators, it is more difficult to know which region requires of greater attention from the supplies and resources.

PHC is the main gateway to the SUS and the coordinator of health care. Therefore, a strong PHC means a health system capable of responding to demands. The present study demonstrated that the results of the PMAQ-AB assessments verified the current scenario and the planning of future actions. The improvement of the program must be part of the process to qualify the functioning. In addition, the program must not regress the data collection format with specificities that qualify the service offered continuously.

## Conclusion

Oral Health Teams from all Brazilian geographic regions showed a reduction in the availability of dental supplies related to amalgam, while an increase of resin composite over time. This could explain the reduction in the number of teams that perform amalgam restorations and the increase of composite resin restorations in the evaluated period. However, regional disparities are still evident, with lower indicators in the North region.
